# On truth and polarity in negation processing: language-specific effects in non-linguistic contexts

**DOI:** 10.3389/fpsyg.2023.1244249

**Published:** 2023-08-16

**Authors:** Norbert Vanek, Haoruo Zhang

**Affiliations:** ^1^School of Cultures, Languages and Linguistics, The University of Auckland, Auckland, New Zealand; ^2^Experimental Research on Central European Languages Lab, Charles University, Prague, Czechia; ^3^College of Foreign Languages, University of Shanghai for Science and Technology, Shanghai, China

**Keywords:** negation processing, linguistic relativity, negative equations, nonverbal cognition, symbolic representation

## Abstract

**Introduction:**

This study examines how negation is processed in a nonverbal context (e.g., when assessing ▲ ≠ ▲) by speakers of a truth-based system like Mandarin and a polarity-based system like English. In a truth-based system, negation may take longer to process because it is typically attached to the negation as a whole (it is not true that triangle does not equal triangle), whereas in polarity-based systems, negation is processed relatively faster because it is attached to just the equation symbol (triangle does not equal triangle), which is processed relatively faster. Our hypothesis was that negation processing routines previously observed for verbal contexts, namely that speakers of Mandarin get slowed down more when processing negative stimuli than positive stimuli compared to speakers of English, also extend to contexts when language use is not obligatory.

**Methods:**

To test this, we asked participants to agree/disagree with equations comprising simple shapes and positive ‘=’ or negative ‘≠’ equation symbols. English speakers showed a response-time advantage over Mandarin speakers in negation conditions. In a separate experiment, we also tested the contribution of equation symbols ‘≠’/‘=’ to the cognitive demands by asking participants to judge shape sameness in symbol-free trials, such as ▲ ■. This comparison allowed us to test whether crosslinguistic differences arise not because of shape congruence judgement but arguably due to negation attachment.

**Results and discussion:**

The effect of the ‘≠’ symbol on shape congruence was language-specific, speeding up English speakers but slowing down Mandarin speakers when the two shapes differed. These findings suggest language-specific processing of negation in negative equations, interpreted as novel support for linguistic relativity.

## Introduction

The ways in which negative yes–no questions are typically answered by speakers of different languages radically vary. In English, the response usually aligns the yes/no part with the verb’s polarity (A*:“Isn’t he feeling well?”* B*:"Yes, he is/No, he’s not”*) when answering. However, in Mandarin, the responses *“shi/shi de”* for *“yes”* and *“bu/bu shi/bu shi de”* for *“no”* typically oppose the verb’s polarity (*“No, he is/Yes, he’s not”*). Mandarin speakers employ a so-called *truth-based* answering system, whereas English speakers use a system known as *polarity-based* (Holmberg, 2015). In the polarity-based system (also known as the positive–negative system), “the choice between *yes* and *no* depends simply on the polarity of the answers” (Huddleston et al., 2002, p. 848). In the truth-based system (or the agree-disagree system) the answers “are determined by agreement with the truth value of the statement which is implied by the question” (Jones,1999; Holmberg, 2015, p. 3). Holmberg (2015, p. 21) reports 44 languages that follow the truth-based system (Japanese) and 49 languages that do not follow the truth-based system (Spanish). This divide is based on the typicality of answers by analyzing native speakers’ intuitions via questionnaires, and it is captured in the online database of Syntactic Structures of the World’s Languages (SSWL). Based on Holmberg’s (2015) categorization, the English language (Germanic, Indo-European) uses the polarity-based system while Mandarin Chinese (Chinese, Sino-Tibetan) belongs to the group of languages following the truth-based system.

There an important difference in the two systems, arises from the distinction in how truth-based and polarity-based languages attach negation (Zhang, 2019; Zhang and Vanek, 2021). Truth-based languages like Mandarin structurally attach negation to the question’s statement, resulting in “*Isn’t he feeling well?*” being transformed to and processed as “*He is not feeling well*.” Conversely, polarity-based languages like English typically attach negation to the question’s polarity, leading to “*Isn’t he feeling well?*” being transformed to and processed as *“Is it the case or not that [he is feeling well].”* Consequently, speakers of truth-based and polarity-based systems have distinct of responding to various statements in negative questions. While English speakers typically respond to the positive statement (Choi, 1991; Holmberg, 2013, 2014, 2015), Mandarin speakers typically respond to the negative statement (Zhang and Vanek, 2021). Here we examine what implications the difference between truth-based and polarity-based systems may have for the processing of negation non-linguistically, in negative equations. Negative equations consist of two geometric shapes and the unequal symbol (▲ ≠ ■).

Another relevant layer of differences between the two systems is that speakers of the truth-based system may be inclined to attach negation more globally, to the whole statement, than speakers of a polarity-based system being inclined to attach negation more locally, for instance to just a word. One piece of evidence for this assumption comes from research on denials, a function of negation. Akiyama (1985, 1992) compared the ways in which Japanese and English children express denials. He instructed 4-year-old Japanese and English children to say the opposite of positive statements such as *A ladybug is large*. While Japanese children preferred to use negative sentences (*A ladybug is not large*), i.e., first verifying the positive statement ‘*A ladybug is large*’ and then reversing its truth value ‘*A ladybug is [not large]*’, English children preferred to use more direct positive sentences (*A ladybug is small*), without reversing the truth value of the positive statement. The observations in Akiyama (1985, 1992) suggest that Japanese and English children follow different routes to process negation, which vary in cognitive demands. Related research with speakers of Hebrew, German and English (Sherman, 1973, 1976; Giora et al., 2004; Kaup et al., 2006) shows that it is easier to process negation attached more locally (*A ladybug is small*) than negation attached more globally (*A ladybug is not large*). Direct crosslinguistic comparisons of a typically truth-based vs. polarity-based system in this respect are currently absent, and so are associated non-linguistic comparisons in domains such as negative equations. Nevertheless, variation in processing different kinds of negations within the polarity-based system is relatively well documented and ready to build on.

In terms of theory, there are two main models that offer contrasting explanations of negation processing, namely the *two-step* and the *one-step* model. In the two-step model, the processing of negation occurs in a sequential manner. Listeners initially form a mental representation of the positive statement and then transition to the representation of the negative statement. Studies supporting this indirect approach have observed increased cognitive demands associated with processing the negative statement compared to the affirmative counterpart (Fischler et al., 1983; Hasson and Glucksberg, 2006; Kaup et al., 2007; Lüdtke et al., 2008; Dudschig and Kaup, 2018). In contrast, the one-step model proposes that the representation of the negative statement is automatic and directly processed, without the need to first represent the positive counterpart (Mayo et al., 2004; Tian et al., 2010; Du et al., 2014; Orenes et al., 2014). The two models diverge in terms of how negation is represented, particularly in relation to the necessity of representing the positive statement first. This distinction in mental representations can be seen as a difference between *iconic* versus *symbolic* representations (Orenes et al., 2014). The two-step model corresponds to iconicity, and more broadly, aligns with the embodiment theory. According to this view, the processing of negation solely entails mental representations that are firmly grounded in the listener’s sensorimotor experiences. When processing a statement like “*The glass is not full*,” individuals first mentally simulate the positive (a full glass) and later move on to mentally simulate the negative (not a full glass). It is currently unknown whether in non-linguistic contexts like negative equations ▲ ≠ ■ individuals also first construct an iconic model of the corresponding affirmative ▲ = ■, and only after this step do they integrate the negation symbol ≠. According to the one-step model, comprehenders mentally simulate the negative state of affairs only (not a full glass). This may be achieved through some symbolic representation of negation, for instance, via a cross as a symbolic marker of falsity (Clark and Chase, 1972). In a non-linguistic context, this would mean a mental representation that directly integrates the negation symbol ≠ without a detour through the affirmative =. Results from studies in the linguistic context are mixed. To assess whether the two-step or the one-step account holds more weight, we next survey what is currently known from experimental research within the polarity-based and the truth-based system.

### Polarity-based and truth-based negation processing: immediate or sequential?

Tian et al. (2010) investigated whether English speakers can process negation directly, in one step. In each trial, English speakers first saw a sentence (*It was Jane who did not cook the spaghetti*), and then they were instructed to verify as quickly as possible whether an object in the following picture was mentioned in the preceding sentence or not. Results showed that English speakers responded significantly faster for matching pictures (uncooked spaghetti for *It was Jane who did not cook the spaghetti*) than mismatching pictures (cooked spaghetti). The match effect was interpreted as evidence that English speakers processed the negative statement in negative sentences immediately, without having to process the positive statement (*It was Jane who cooked the spaghetti*) first. An alternative explanation for the match effect was provided as well, namely that cleft sentences represent a specific kind of negation where the negative predicate is not emphasized. Thus, participants may not have fully processed the negation in this condition, leaving open the possibility that faster response speed to the matching images could have been driven by image-related properties. Tests with different negation types could be more informative in this respect. In a related neurophysiological study, Nieuwland and Kuperberg (2008) measured ERPs of English speakers while they processed what the authors called the ‘pragmatically licensed negation’ (*With proper equipment, scuba-diving is not very dangerous*). A greater N400 appeared in both positive and negative sentences when these sentences were false than when they were true. These results led Nieuwland and Kuperberg (2008) to reason that English speakers process pragmatically felicitous negation immediately, just as they process positive statements. To sum up, Nieuwland and Kuperberg (2008) and Tian et al. (2010) provide support for the idea that English speakers can process negation immediately, in one step.

Evidence for a one-step access to negative statements also comes from speakers of polarity-based languages other than English, including Spanish and Italian (Holmberg, 2015). To explore the processing of negation in Spanish speakers, Orenes et al. (2014) examined eye fixations on four colored items when participants listened to a negative sentence (*The figure was not red*). Before the target sentence, Spanish speakers first heard a contextual sentence (*The figure could be red or green*). The results showed that Spanish speakers focused more on the alternative-to-the-negated color (i.e., green). The researchers reasoned that Spanish speakers, representatives of a polarity-based language other than English, can process negation immediately, in a single step. In another negation processing study using functional magnetic resonance imaging (fMRI), Tettamanti et al. (2008) measured the brain activity of Italian speakers when they processed positive/negative sentences (*push/not push the button*). The results showed that both statement types activated the brain’s motor regions, but the activation level was significantly lower for sentences that were negative compared to sentences that were positive. These results may suggest that speakers of Italian, another polarity-based language, process negation immediately. The rationale is that had Italian speakers processed negation sequentially in two steps, negative sentences would first have activated the brain’s motor regions to a similar extent than positive sentences, and activation would then have faded off (a step) later. Nonetheless, the authors did not interpret these results as direct evidence for an immediate processing of negation in negative sentences because the temporal resolution of fMRI data may not be fine enough to capture key temporal distinctions to differentiate between one-step and two-step negation processing.

While accumulating evidence from studies with a range of paradigms suggests that speakers of a polarity-based system like English can process negation immediately in a single step, little is known about the truth-based system in this respect. To test whether a single-step processing option is available or not to speakers of the less direct truth-based system, Zhang and Vanek (2021) asked English and Mandarin speakers to formulate answers to positive/negative yes–no questions. One negation condition was negative-same (the question was *Didn’t he steal a duck?* and the given statement was *He stole a duck*), in which the English group predominantly answered “yes” and the Mandarin group answered *bu (shi de)* “no.” The other negation condition was negative-different (the question was *Didn’t he steal a chicken?* and the given statement was *He stole a duck*), in which the English group predominantly answered “no” and the Mandarin group answered *shi (de)* “yes.” On top of the robust crosslinguistic difference in response type with the English following the polarity-based system and the Mandarin group following the truth-based system, English speakers exhibited a reaction-time advantage over Mandarin speakers, suggesting that for Mandarin speakers may need an extra/longer step. This pattern was mirrored in a separate comprehension experiment, in which participants were asked to respond to the same kinds of questions with speeded Y/N button presses instead of formulating yes–no answers. These findings prompted the interpretation that polarity-based English guides its speakers to respond to the positive statement (*He stole a duck*) of a negative question (Choi, 1991; Holmberg, 2013, 2014, 2015), whereas truth-based Mandarin guides its speakers to respond to the negative statement (*He did not steel a duck*) of a negative question (Lu, 2005; Huang and Liao, 2007; Holmberg, 2015). The present study is motivated by these crosslinguistic differences. If speakers of truth-based and polarity-based languages habitually process negation in negative questions differently, it may lead to different processing routines. As a result, language-specific processing routines may persist even when linguistic cues are removed. Empirical evidence for this idea is abundant in many domains, yet, it has not been attested in the domain of negation.

### Processing consequences of more local vs. more global negation attachment

Linguistic encoding of negation more locally is referred to as lexical negation (*The umbrella is closed*) and that more globally, at the sentential level, as negative particle negation (*The umbrella is not open*). The latter arguably comes with greater difficulty to process than the former. Support can be found already as far back as in Sherman (1973, 1976), who tested response times of English speakers when they verified positive sentences (*She was happy*) and negative sentences with implicit negation (*She was sad*), lexical negation (*She was unhappy*), and a negative particle (*She was not happy*). They found that it took English speakers a similar amount of time to process negation at the lexical level (*unhappy* and *sad*). However, it took English speakers significantly shorter to process the lexical negation *unhappy* (310 ms longer than positive sentences) than the negative particle *not* (520 ms longer than positive sentences). In Sherman’s view, negation at the lexical level such as *unhappy* provides a shortcut for English speakers to process negation because the reversal of the meaning of a word, more locally, is easier than that of a sentence, more globally. These findings point to increased processing demands when negation is attached to a statement (more globally) compared to when negation is attached to a word (more locally). However, critics could argue that the findings may be attributed to the difference in semantics rather than negation type. In lexical negation, *unhappy* is a synonym for *sad*, so the mental state is clear, whereas in particle negation, *not happy* could mean any emotion other than happiness, so the mental state is underspecified. This difference is problematic for firm conclusions, and it highlights the need to work with designs that eliminate potential confounds (in this case semantic underspecification) if the aim is to test whether processing speed varies as a function of negation attachment.

The idea of increased processing demands when negation attachment is more global rather than more local aligns with Carpenter and Just’s (1975) *Constituent Comparison Model* (CCM). According to this model, the expression of negation is closely associated with its scope. The smaller the scope of negation, the less difficult it is to process. To test this prediction, the researchers conducted a sentence-picture verification task. English speakers were asked to verify pictures paired with sentence including negative markers with large scope (*It is not true that the dots are red*) and small scope (*It’s true that the dots aren’t red*). It took English speakers 500 ms longer to process negative particles with large scope than with small scope. Following the CCM, variation in the scope of negation in Mandarin (more typically statement-attached) vs. English (more typically word-attached) could lead to language-specific processing patterns. A more local attachment of negation being easier to process was also found in Hebrew. Giora et al. (2004) designed a sentence probing task to investigate the processing of negation in different forms. Two types of negation were used, i.e., the negative particle (*The instrument is not sharp*) and implicit negation (*The instrument is blunt*). Participants were instructed to read the sentence and then judge whether the following word (semantically related/unrelated, such as *piercing/leaving*) is a real word or a not. The results showed that sentences with the negative particle (*not sharp*) facilitated the processing of *piercing*. In contrast, implicit negation (*blunt*) did not help process piercing more efficiently. The varied processing speed observed in *not sharp* and *blunt* is interpreted as further support for the idea of greater difficulty in attaching negation more globally to a statement compared to attaching negation more locally to a word. Further support for this idea can be found in Kaup et al. (2006), who also documented slowdowns induced by more global attachment of negation compared to negation at the lexical level. They showed German speakers a sentence with implicit negation/the negative particle (*The umbrella is closed/not open*), after which the participants saw a picture matching/mismatching the state of the object depicted in the previous sentence (a closed umbrella/an open umbrella for *The umbrella is not open*). The task was to name the object in the picture as quickly as possible. Intervals between the sentence and the corresponding picture were either short (750 ms) or long (1,500 ms). With a short interval, a match effect only emerged for negation at the lexical level (i.e., faster RT for a closed umbrella than an open umbrella following *The umbrella is closed*). However, only with a longer interval of 1,500 ms between the sentence and the picture was a match effect found for the negative particle. The researchers interpreted the longer time needed to observe a match effect for the negative particle as more cognitively demanding negation processed at a later phase. In this study, we build on the findings about greater difficulty reported in attaching negation more globally than more locally (Sherman, 1973, 1976; Akiyama, 1979; Choi, 1991; Giora et al., 2004; Kaup et al., 2006). We expand previous research on negation processing in Mandarin vs. English a verbal context (Zhang and Vanek, 2021), which suggests that Mandarin as a truth-based language system entrains routines of a more global negation attachment, while English as a polarity-based language system entrains routines of a more local negation attachment. Our aim is to examine whether the effect of this crosslinguistic difference on processing routines percolates through to non-linguistic contexts.

## Experiment 1

The theory of linguistic relativity predicts that if English and Mandarin speakers follow language-entrained routines to process negative yes–no questions in a verbal context, they would also be influenced by these routines in a nonverbal context. In Experiment 1 we tested this hypothesis by examining the reaction times of English and Mandarin speakers during negative equation verification. The negative equation verification paradigm is used here as a nonverbal analogue to negative yes–no questions (Zhang and Vanek, 2021). We presented participants with two geometric shapes put into an equation. Half of the trials involved the equal symbol (‘=’), and half involved the unequal symbol (▲ ≠ ■). If Mandarin speakers are more likely than English speakers to attach negation to the statement ‘triangle does not equal square’ also in a context without overt verbalization, then Mandarin speakers would answer negative equations relatively more slowly than English speakers. Alternatively, the null hypothesis is that English and Mandarin speakers process negative equations in the same way, in which case no crosslinguistic contrast in reaction times would be expected.

### Methods

#### Participants

Forty participants took part, including 20 native English speakers (17 females; all students at a UK university; mean age 19.5, range 5 years) and 20 native Mandarin speakers (20 females, all students at a college of preschool education in China; mean age 20.4, range 2 years). All participants were right-handed and reported no fluency in any language other than their L1. The choice of the targeted sample size followed previous studies using similar group sizes in comparable tasks with positive/negative conditions (Choi, 1991; Lüdtke et al., 2008). The same participants also took part in negation experiments with overt verbalization (Zhang and Vanek, 2021). An effort was made to ensure the nonverbal experiment always came first to avoid potential self-priming and training-induced biases.

#### Stimuli

**Figure 1:**
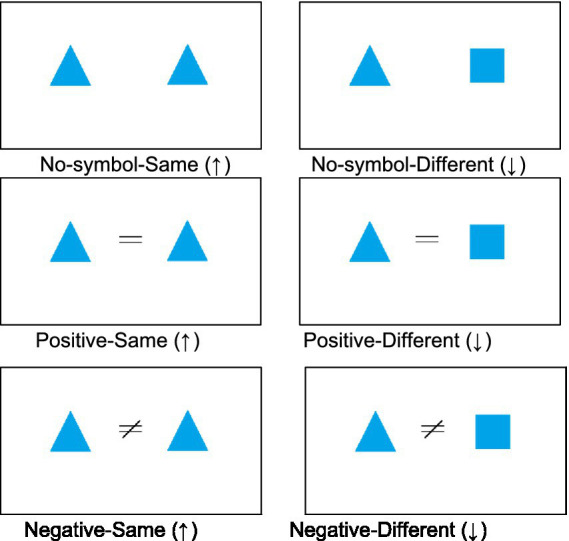
Example stimuli showing the six conditions in Experiment 2 and the corresponding correct responses in brackets.

Thirteen blue-colored shapes and two symbols (i.e., the equal symbol ‘=’ and the unequal symbol ‘≠’) were used to form 24 distinct equations (Figure 1). The lengths of the two symbols ‘=’ and ‘≠’ on the screen was 3.2 cm. The height of each equal symbols ‘=’ was 0.9 cm and that of the unequal symbol ‘≠’ was 2.6 cm. The heights of the shapes ranged from 4.4 cm (trapeziums) to 6.2 cm (annuluses). The distance between the centers of the two shapes that appeared simultaneously in each trial was 16.5 cm. The resolution ratio of the screen was 1,366 × 768 pixels. The shapes and the symbols appeared centered on the screen. The combinations of shapes and symbols were divided into four conditions based on the polarity of the equation symbol (positive/negative) and the sameness of the shapes (positive-same, positive-different, negative-same and negative-different), as shown in Figure 1 with the corresponding correct arrow presses.

#### Procedure

The stimuli were presented using E-Prime 2.0. on a laptop with a 15.6-inch screen. Participants were tested individually. They were asked to carefully read the instructions displayed on the screen at the beginning of the test. They were informed that they would see “some equations represented by shapes and symbols.” Their task was to “agree or disagree with these equations as fast and accurately as possible.” Participants were asked to press ‘↑’ on the keyboard when they agree with an equation and ‘↓’ when they disagree with it. They were instructed to use their right index finger to press keys throughout the experiment and rest their index finger between the two keys ‘↑’ and ‘↓’ when they were not pressing any keys. This procedure helped to avoid finger-switching costs, and also helped to minimize possible pre-existing temporo-spatial metaphorical associations such as ‘left is earlier’ and ‘right is later,’ which were shown to be associated with the speed of button presses (Fuhrman et al., 2011). For both positive and negative conditions the button/correctness association was counterbalanced. That is, for 50% of trials ‘↑’ was the correct button press, and for the other 50% of trials ‘↓’ was correct. The test started with a brief training session including 4 trials (i.e., 2 same/different shapes × 2 types of symbols) before the experimental session. Participants were informed that if they make a mistake during the trials, they should not stop but continue with the following equation.

During the experimental session, the participants saw two blue shapes and an equation symbol (either the equal symbol ‘=’ or the unequal symbol ‘≠’) on a white background in each trial. When a key ‘↑’ or ‘↓’ was pressed by the participant, the computerized task would automatically display a blank screen (1,000 ms) before showing the next trial. The order of the trials was semi-randomized to ensure that the same condition would not appear more than twice consecutively. Each response and reaction time was recorded. Whenever a sustained pause was observed as a result of a participant’s mistake, this was noted down and the data for that trial was eliminated from subsequent analyses [i.e., 1 English (0.2% of total) and 3 Mandarin (0.6% of total) data entries]. All procedures were approved by the Ethics Committee of the Department of Education, University of York.

### Results

**Figure 2:**
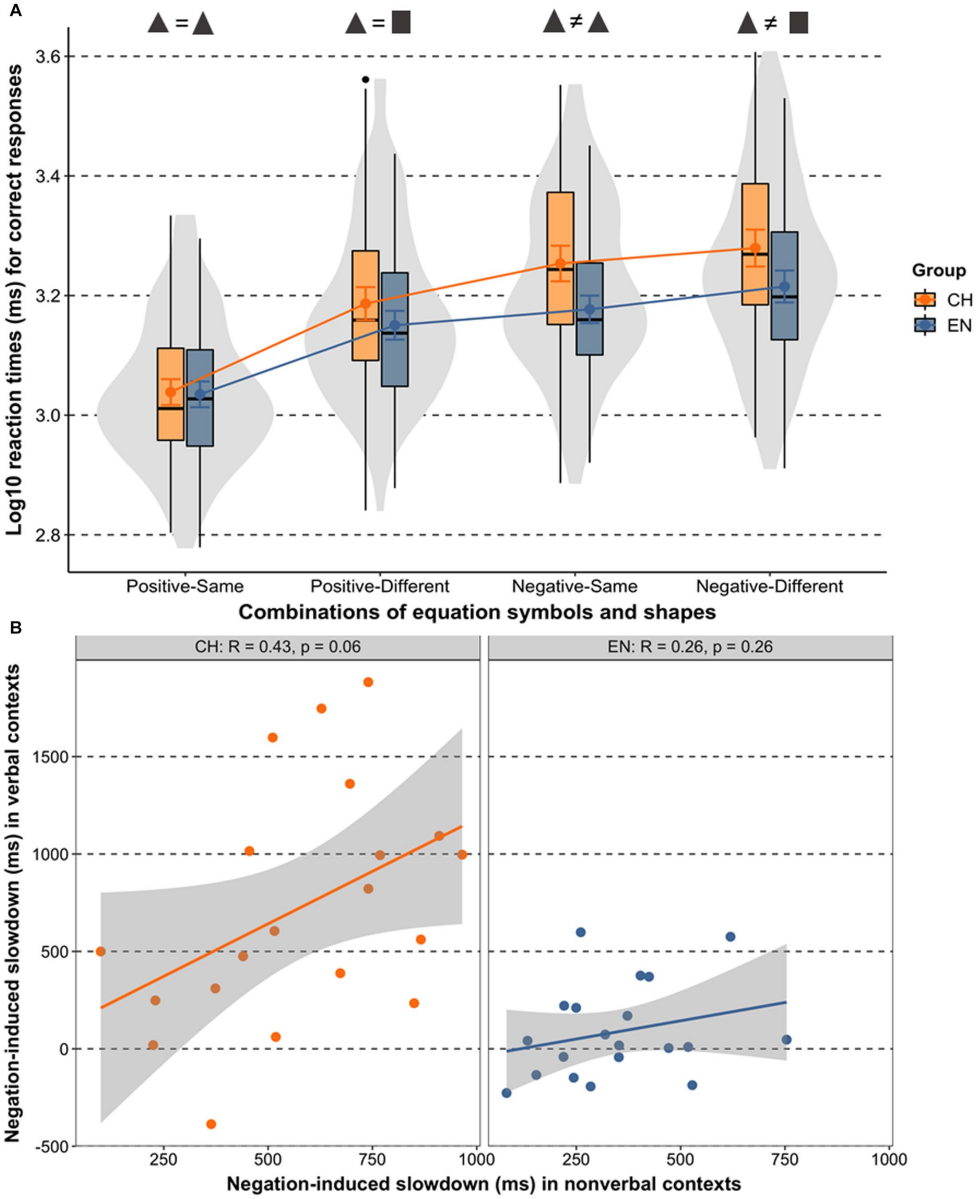
**(A)** Log-transformed response times in Experiment 1 by group and condition. Colored dots show the means, the slopes of the connecting lines express the relative slowdown between conditions, error bars around the means are 95% confidence intervals, box and violin plots show data distributions. **(B)** Correlation plots display the relationship between participants’ negation-induced slowdowns in Experiment 1 and the same participants’ slowdowns in verbal negation reported in Zhang and Vanek (2021) (data available from https://osf.io/x4536/).

We first considered response speed differences between positive and negative equations. The mean RT of English speakers verifying negative equations was 1,647 ms (SD = 535) and that of Mandarin speakers was 1967 ms (SD = 706). The mean RT of English speakers verifying positive equations was 1,304 ms (SD = 446) and that of Mandarin speakers was 1,376 ms (SD = 559). These results show that it took English and Mandarin speakers longer to verify positive equations when the shapes in those equations were different (▲ = ■) than when the shapes were the same (▲ = ▲). Analogously, it also took English and Mandarin speakers longer to verify a negative equation when the shapes in an equation were different (▲ ≠ ■) than when the shapes were the same (▲ ≠ ▲). Initial response accuracy checks showed that the English participants correctly answered 96.04% and the Mandarin participants 90.83% of the trials. Only RTs of correct responses were analyzed. To eliminate outliers, 13 English (2.7% of total) and 12 Mandarin (2.5% of total) data entries were more than 2.5 standard deviations away from the group mean in each condition and they were replaced by the cut-offs (group mean +/− 2.5 SDs). Figure 2A plots the data distribution per group and condition.

To answer our research questions, we built mixed-effect regression models using the lme4 package (Baayen et al., 2008) in R (Version 4.1.1; R Development Core Team, 2021). Our fixed effect factors were *Language Group* (English/Mandarin), *Equation symbol* (positive/negative) and *Sameness* (same/different) and the random effect factors were *Participant* and *Item*. The model, as shown in Table 1, included all possible random effects (Barr et al., 2013), with random slopes over equation symbol, sameness and their interaction by participant and random slopes over equation symbol by item [RT ~ equationsymbol * sameness * group + (1 + equationsymbol * sameness | participant) + (1 + equationsymbol | item)]. To explore the effect of equation type, we next built a reduced model without *Equation symbol.* A comparison of the reduced model with the full model showed that the presence of *Equation symbol* significantly increased the model fit, χ2(13) = 84.95, *p* < 0.001, confirming that participants answered negative questions significantly more slowly than positive questions.

**Table 1:** Coefficients for a mixed effects model fitted to the RTs of English and Mandarin speakers in equation verifications in Experiment 1.

Fixed effects	Estimate	SE	*t* value	*p*
Intercept	2028.70	106.89	18.98	<0.001**
Equationsymbol (pos)	−386.95	79.80	−4.85	<0.001**
Sameness (same)	−135.38	96.93	−1.40	0.171
Group (EN)	−292.61	147.52	−3.18	0.054
Equationsymbol (pos) × Sameness (same)	−369.44	119.23	−3.10	0.004*
Equationsymbol (pos) × Group (EN)	134.53	97.70	1.38	0.173
Sameness (same) × Group (EN)	−35.72	128.62	−0.28	0.783
Equationsymbol (pos) × Sameness (same) × Group (EN)	181.62	148.81	1.22	0.229

Random effects	Variance	SD
Participant	1,800,009	424.27
Item	2,840	102.51

A single asterisk * indicates *p* < 0.05 and double asterisks ** indicate *p* < 0.001.

Then, proceeding with a forward variable selection with focus on the negative equations only, we compared a model including *Language Group* with a reduced model without *Language Group* to check if RTs for negative questions are group-specific. Critically, this comparison confirmed a significant contribution of *Language Group* to the variation in responses to negative equations, χ2(2) = 8.32 *p* = 0.002. We did the same to compare whether there was a difference between groups in positive-same and positive-different conditions, but found that *Language Group* was not a significant factor, χ2(2) = 2.42 *p* = 0.298. As the last statistical step, we ran Tukey-adjusted pairwise comparisons to test how group responses differ for various types of negative equations. While in the negative-different condition the difference between the Mandarin and English speakers’ response speed only approached significance (estimate = 289, *SE* = 147, *t* = 1.96, *p* = 0.057), the Mandarin speakers were significantly slower in the negative same condition than the English speakers (estimate = 328, *SE* = 108, *t* = 3.03, *p* = 0.004). These results show that the negation-induced slowdown was relatively more robust for Mandarin speakers than it was for the English speakers, as predicted, and that verifying negative equations combined with the same shapes presented the main between-group difference. The full dataset with RTs per participant and condition as well as the annotated R codes and corresponding model outputs are available on the project website https://osf.io/qmgj2/.

### Discussion

Experiment 1 set out to investigate whether English and Mandarin speakers process negative equations in language-specific ways. Crosslinguistic differences in reaction times suggest that it is indeed the case. Mandarin speakers, in comparison to English speakers, exhibited a greater slowdown in response speed to negative equations compared with positive equations. We attribute this increased slowdown in Mandarin speakers to a stronger tendency than in English speakers to follow the truth-based system and attach negation to the equation as a whole, rather than to follow the polarity-based system and attach negation to just the equation symbol. Our claim is that, during verification, ▲ ≠ ▲ in Mandarin speakers was processed as ‘[is it true or not that] triangle does not equal triangle?’ The habitual answer to this type of truth-based questions or verifications comes with the attachment of negation to the whole statement ([it is not true that] triangle does not equal triangle). Unlike in Mandarin speakers, the comparatively smaller slowdowns when English speakers process negative equations suggest that they followed the polarity-based system. This would mean that they attached negation to the equation symbol ≠ rather than to the equation as a whole ▲ ≠ ▲. The proposed interpretation of the different patterns in English and Mandarin speakers’ processing of negative equations aligns with earlier findings from verbal experiments that point to a greater difficulty to process negation when it is attached to a whole statement than to a single word (Sherman, 1973, 1976; Giora et al., 2004; Kaup et al., 2006). In this experiment, the slowdown in Mandarin speakers was 248 ms greater than that in English speakers, closely approximating the earlier observed 210 ms difference reported for the statement-versus-word-based attachment of negation (Sherman, 1973, 1976). It is important to note that even though negative equations do not explicitly capture the linguistic operator of negation, the observed between-group differences in negation-induced slowdowns are indicative of language-specific routines in negation processing.

One might ponder whether Mandarin speakers exhibit greater negation-induced slowdowns only when the going gets tough, that is, when equation verification is combined with shape sameness judgements. To investigate whether English and Mandarin speakers’ negative equation processing differs just because of a shape mismatch or primarily because of language-specific attachment of negation in equation verification, we designed Experiment 2 with shape sameness judgement and negation attachment disentangled.

## Experiment 2

We designed Experiment 2 to test the contribution of equation symbols ‘≠’/‘=’ to the time it takes English and Mandarin participants to make shape sameness judgements. To this end, participants judged whether two shapes were the same in the presence or absence of equation symbols. The instructions focused on shape sameness, the ‘≠’/‘=’ symbols were irrelevant for task completion. With the symbols present we aimed to check whether ‘≠’/‘=’ between the two shapes facilitates or hinders response speed depending on shape sameness. Namely, if congruence plays a role, one could expect to see facilitation in conditions where ‘=’ is combined with the same shapes and ‘≠’ with different shapes, while a slowdown is more likely in conditions where ‘≠’ is combined with the same shapes and ‘=’ with different shapes. Across groups, if English speakers have a stronger tendency to attach negation to the equation symbol, and Mandarin speakers are more likely to attach negation to the whole equation, then stronger facilitation/slowdown of the ‘≠’/‘=’ symbols would emerge in the English group than in the Mandarin group. Symbol-free trials, such as ▲ ■, were included in this experiment as controls to establish if crosslinguistic differences arise not because of sameness judgement but due to negation attachment. If this holds, one would expect to find no crosslinguistic differences in the symbol-free conditions.

### Participants

Forty participants other than those in Experiment 1 were recruited for Experiment 2. In an effort to ensure consistency across experiments, the inclusion criteria for Experiment 1 were identical with those in Experiment 2 regarding L1 dominance, age, tertiary level student status and handedness. Twenty were native English speakers (18 females; all students at a UK university; mean age 20.3, range 5 years) and 20 were native Mandarin speakers (20 females, all students at a college of preschool education in China; mean age 21.0, range 3 years). All participants were right-handed and reported no fluency in any language other than their L1.

### Materials

Eight blue-colored shapes, randomly selected from the 13 shapes used in Experiment 1, and two equation symbols ‘=’ and ‘≠’ were used to form 24 distinct equations and 12 pairs of shapes without symbols (see Figure 1). The combinations of symbols and shapes formed six types of conditions (i.e., no-symbol-same, no-symbol-different, positive-same, positive-different, negative-same and negative-different) as shown in Figure 1.

### Procedure

The procedure of Experiment 2 was similar to Experiment 1, with the only difference that participants were instructed to judge whether the two shapes “are the same or not the same.” The task was to press ‘↑’ on the keyboard when the two shapes were the same, and ‘↓’ when they were not the same. Participants’ responses and RTs were collected. When a sustained pause was observed because a participant made a mistake, this was noted down and the data entry for the trial during which the pause happened was eliminated from subsequent analyses (2 cases in the English dataset (0.3% of total) were eliminated this way).

### Results

**Figure 3:**
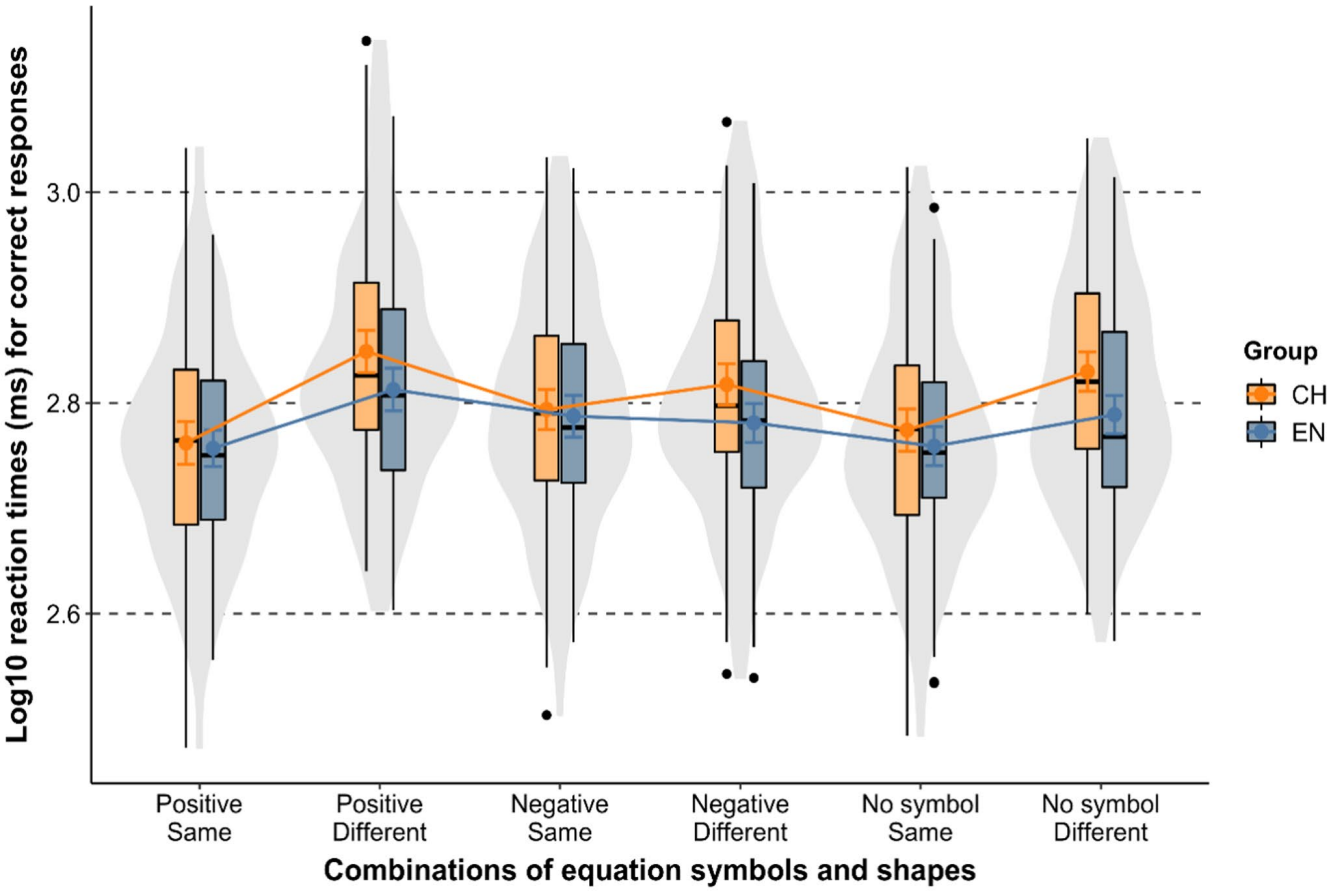
Log-transformed response times in Experiment 2 by group and condition. Colored dots show the means, the slopes of the connecting lines express the relative processing difficulty of different conditions, error bars around the means are 95% confidence intervals, box and violin plots show data distributions.

The response accuracy of English participants was 97. 08% and that of Mandarin participants was 97.64%. Only RTs of correct responses were included in the analyses. There were 16 English (2.22% of total correct responses) and 12 Mandarin outliers (1.67% of total correct responses), whose RTs were more than 2.5 standard deviations away from the mean of the group in each condition. The data distribution per group and condition is plotted in Figure 3. The mean RT of English speakers to correctly identify same shapes was 601 ms (SD = 144) and that of Mandarin speakers was 617 ms (SD = 156), while the mean RT of English speakers to correctly identify different shapes was 641 ms (SD = 161) and that of Mandarin speakers was 701 ms (SD = 183). Characteristic of both groups was that the ‘≠’ symbol facilitated response speed in the negative-different condition (*M*EN = 621, SD = 148; *M*CH = 678, SD = 175) compared to the no-symbol-different condition (*M*EN = 632, SD = 152; *M*CH = 694, SD = 164), and it slowed down response speed in the negative-same condition (*M*EN = 632, SD = 160; *M*CH = 640, SD = 151) compared to the no-symbol-same condition (*M*EN = 590, SD = 141; *M*CH = 614, SD = 160). However, facilitation and slowdown strength also differed in an important respect across groups. With the ‘≠’ symbol present, English speakers responded faster when the two shapes were different (*M* = 621, SD = 148) compared to when they were the same (*M* = 632, SD = 160); and in contrast, Mandarin speakers reacted slower when the two shapes were different (*M* = 678, SD = 175) than when they were the same (*M* = 640, SD = 151).

Next, we built three models to compare how English and Mandarin speakers process different types of negative equations (critical condition), shapes without equation symbols (first control), and positive equations (second control). In each model, *Sameness* and *Language Group* were used as fixed effects factors, and *Participan*t and *Item* as random effect factors. Interactions between the fixed effects factors were also tested, and all possible random effects were included to keep the models maximal, using the formula RT ~ 1 + sameness * group + (1 + sameness | participant) + (1 + sameness| item). Care was taken in the adopted analytical approach to make meaningful comparisons of congruence effects in different conditions. We examined whether ‘≠’/‘=’ between the two shapes facilitates or hinders response speed depending on shape sameness in the two groups. Three models were built since congruence involves different shape-symbol combinations across conditions. We first focused on the negative symbol, and tested if ‘≠’ influences shape sameness judgements in language-specific ways. Second, we zoomed in on the positive symbol, and tested if ‘=’ with same shapes speeds RTs up and with different shapes slow RTs down irrespective of language group. And third, we checked whether in the absence of symbols same shapes get processed faster than different shapes irrespective of language group. The model output is shown in Table 2, and the dataset with RTs per participant and condition as well as the annotated R codes are available on the project website https://osf.io/qmgj2/.

**Table 2:** Coefficients for the mixed effects models fitted to the RTs of English and Mandarin speakers in Experiment 2.

Negative symbol (critical)				
Fixed effects	Estimate	SE	*t* value	*p*
Intercept	678.28	31.85	21.30	<0.001**
Sameness (same)	−37.59	23.73	−1.58	0.145
Group (EN)	−59.62	37.23	−1.60	0.117
Sameness (same) × Group (EN)	47.97	21.70	2.21	0.028*
Random effects	Variance		SD	
Participant	11,585		107.64	
Item	1922		43.85	
No symbol (control)				
Fixed effects	Estimate	SE	*t* value	*p*
Intercept	693.54	31.46	22.05	< 0.001**
Sameness (same)	−79.41	27.07	−2.93	< 0.013*
Group (EN)	−61.38	35.88	−1.71	0.095
Sameness (same) × Group (EN)	36.28	24.97	1.45	0.154
Random effects	Variance		SD	
Participant	10,951		104.65	
Item	2072		45.52	
Positive symbol (control)				
Fixed effects	Estimate	SE	*t* value	*p*
Intercept	730.17	44.40	16.45	<0.001**
Sameness (same)	−133.08	46.49	−2.86	<0.018*
Group (EN)	−60.69	37.90	−1.60	0.117
Sameness (same) × Group (EN)	47.81	34.36	1.39	0.171
Random effects	Variance		SD	
Participant	11,935		109.25	
Item	7,523		86.74	

A single asterisk * indicates *p* < 0.05 and double asterisks ** indicate *p* < 0.001.

None of the three models returned a significant effect of *Group*, which points to the response speed of English and Mandarin speakers being similar across all three conditions, i.e., with no equation symbol, with ‘=’ as well as with ‘≠’. Importantly, in the negative equations, a significant interaction was found between *Sameness* and *Group,* showing that the reaction time pattern was significantly different in English and Mandarin speakers, as predicted. This result statistically confirmed that the ‘≠’ symbol speeded up English speakers’ reactions when the two shapes were different compared to when they were the same, and, that it slowed down Mandarin speakers’ reactions when the two shapes were different compared to when they were the same. Such a between-group asymmetry in the reaction time pattern only surfaced in the critical ‘negative symbol’ condition. No significant interaction was found between *Sameness* and *Group* in either of the control conditions, as predicted. This absence of a significant interaction in both the ‘no symbol’ and the ‘positive symbol’ control conditions shows that English and Mandarin speakers were slowed down to a similar extent when the two shapes were different compared to when the shapes were the same.

### Discussion

The language-specific effect of the ‘≠’ symbol on shape congruence judgements found in Experiment 2 provides further empirical support for the claim that Mandarin speakers are more likely than English speakers to follow the truth-based system and attach negation to the whole statement. For both groups, the ‘≠’ symbol facilitated response speed in the negative-different condition compared to the no-symbol-different condition, and it slowed down response speed in the negative-same condition compared to the no-symbol-same condition. However, there was a crosslinguistic difference in the strength of facilitation and slowdown. In the condition with ‘≠’, English speakers responded faster when the two shapes were different compared to when they were the same. Conversely, Mandarin speakers responded slower in the condition with ‘≠’ when the two shapes were different compared to when they were the same. Our explanation for the observed asymmetry is that English speakers are likely to follow the polarity-based system and attach negation to the equation symbol while Mandarin speakers are more likely to follow the truth-based system and attach negation to the whole equation. To illustrate this difference, when judging shape sameness for the stimulus ‘triangle-unequal-square,’ English speakers may be more strongly guided by the polarity-based system to process it as ‘does triangle not equal square?’, while Mandarin may be more strongly guided by the truth-based system to process it as ‘is it true or not that triangle does not equal square?’. This explanation aligns with findings showing that negation attached to shorter linguistic units, such as a word/verb, facilitates recognition speed of the negative state of affairs more strongly than negation attached to longer linguistic units such as a whole statement (Giora et al., 2004; Kaup et al., 2006).

The crosslinguistic difference in the processing of the ‘≠’ symbol in negative equations found in Experiment 2 cannot be explained by possible domain-general cognitive differences in English and Mandarin speakers. First, there was no between-group contrast found in the control conditions. That is, when there was no equation symbol as well as when the positive ‘=’ symbol were co-presented with shapes, no between-group contrast was found for reaction time patterns. This result indicates that the cognitive abilities of English and Mandarin participants tested were comparable. Second, unlike in Experiment 1 where negative equations were more difficult than positive equations by design, in Experiment 2 the difficulty of the critical condition and the control conditions were kept the same by asking the participants to judge the sameness of shape pairs irrespective of the equation symbol. With this feature controlled in the design, it was possible to ascertain that the observed language-specificity in the processing of the ‘≠’ symbol cannot simply be attributed to the increased difficulty when the task is to verify negative equations compared to when the task is to judge shape sameness. Instead, language-specific attachment of negation can better account for the response time variations when English and Mandarin speakers process negative equations.

## General discussion

### Truth-based vs. polarity-based processing of negative equations

Responses to negative questions in English and Mandarin substantially differ. This could be because Mandarin speakers typically attach negation to the statement of the question while English speakers typically attach negation to the polarity of the question. While processing consequences of this differences in verbal contexts were captured in previous studies (Zhang and Vanek, 2021), here we were intrigued by the possibility that the impact of language on how negation is processed may extend to situations when language is not needed. We examined the speed with which negative equations are verified. Our results revealed that it took Mandarin speakers significantly longer than English speakers to process negative equations than positive equations. We interpret these results as support for the hypothesis that language-specific routines in processing negative questions extend to a nonverbal context of processing negative equations.

Such routines can be linked to typological differences in answering system. In Mandarin, following a truth-based system means that speakers attach negation to the whole statement. This idea received support from previous studies looking at yes/no answers to negative questions (Akiyama, 1979; Choi, 1991; Holmberg, 2015). To illustrate, when Mandarin native speakers process a negative question (*Isn’t the glass full?*), they typically attach negation to whole statement of the question ‘the glass is not full’. In this scenario, Mandarin speakers have to keep in mind the negative statement ‘Is it true that [the glass is not full]‘and compute the truth value over that statement, which is arguably more demanding than computing the truth value of the corresponding positive statement. If a truth-based system directs its speakers to attach negation to the whole statement, then it is not surprising to find that Mandarin native speakers also attach negation to the whole equation when they process negative equations. The idea that attachment of negation processing can be routinised reaches back to Akiyama (1985, 1992).

Unlike in Mandarin, in English speakers typically follow the polarity-based system, which means they respond to the positive statement (*The glass is full*) of a negative question (*Isn’t the glass full?*) (Choi, 1991; Holmberg, 2013, 2014, 2015). When English speakers process a negative question like *Isn’t the glass full?*, attaching negation to the polarity of the question would mean processing negation as *Is it the case that [the glass is full]*. In a non-linguistic context, when English speakers were confronted with a truth-based Mandarin-like way to agree/disagree with negative equations, they were faster as they were not constrained by their language as much as Mandarin speakers to attach negation to the whole statement. Instead, English speakers were more likely to attach negation to the equation symbol rather than to the equation as a whole, and the response times show it is easier to process negation locally attached to a single word/symbol than to a whole statement/equation (Sherman, 1973, 1976; Giora et al., 2004; Kaup et al., 2006). To illustrate negation attachment to the equation symbol, English speakers may be more inclined to process the negative equation ‘triangle-unequal-square’ as ‘the triangle and the square are unequal’.

### On embodiment in negation processing

This study’s main contribution to theory is through providing new evidence that aligns with the two-step negation processing model (Kaup et al., 2006, 2007) and is consistent with the embodied cognition view. This view denies the possibility that something that *is not*, such as a negative equation symbol, can form a part of one’s mental representation. Instead, complete grounding in sensorimotor experience is necessary to mentally represent a state of affairs because comprehension is achieved through action simulations, which in a sentence are expressed by the main verb (Barsalou, 1999; Zwaan, 2016). Following this theory, negation processing needs to take two steps. In them, individuals first simulate the affirmative (a glass is full) and then move on to the corresponding negative (a glass is not full). Negation-induced slowdowns observed in the present experiments suggest that comprehenders start with the simulation of the affirmative (triangle equals square) and only then proceed to the simulation of the negated alternative (triangle does not equal square). We interpret the extra time costs consistently observed when both groups of participants processed negative compared to positive equations as a signal that comprehenders first construct a representation of the negated argument and subsequently reject it in favor of the alternative. In other words, added cognitive demands exhibited as slower reaction times to negative equations indicate that to understand ▲ ≠ ■, individuals build an iconic mental model of the corresponding affirmative ▲ = ■ and then integrate the negation symbol ≠. This interpretation aligns well with related studies on the cognitive mapping of negation which suggest that negation is not immediately integrated (Kaup et al., 2006; Lüdtke et al., 2008; de Vega et al., 2016; Beltrán et al., 2018).

The observed crosslinguistic phenomena help further refine the embodied cognition account. More robust negation-induced slowdowns in the Mandarin group than in the English group during equation verifications in Experiment 1 show that that negation integration is delayed in language-specific ways. Following the view of language-specificity in negation attachment, Mandarin speakers integrate negation later than English speakers as a result of greater cognitive demands linked to the truth-based system than to the polarity-based system. Namely, swapping the representation of the negated argument for the corresponding alternative takes longer for Chinese speakers because in a truth-based system negation is attached to the whole equation, i.e., more globally. Comparatively less time is needed to swap representations for English speakers since a polarity-based system requires attachment of negation to just the ≠ symbol, i.e., more locally. Findings from shape sameness judgments in Experiment 2 provide further insights into the language-specific modulations of the cognitive effort associated with negation processing. Let us revisit the inter-group asymmetry observed in shape sameness judgements. When the two shapes were different, the ‘≠’ symbol facilitated response speed for English speakers, but it slowed response times down for Mandarin speakers compared to where the two shapes were the same. These results dovetail with the view of local vs. global attachment of negation (Carpenter and Just, 1975; Zhang et al., 2022) and its implications for cognitive processing within the embodiment theory. In a polarity-based system, assumed to represent the English group, facilitation may be attributed to the quick (local) check of congruency between the unequal shapes and the unequal symbol. However, in a truth-based system, representative of the Mandarin group, an overall slowdown may be attributable to the more holistic (global) check involving an incongruency from a truth-based perspective (it is not true that the two shapes are the same, but it is true that triangle does not equal square). Crosslinguistic differences in negation processing can thus further enrich the two-step simulation theory and the embodiment view. Nonetheless, it should be noted that the prospect of negation being embodied is still very much a target of resonant debate and invites further research.

### Avenues for future inquiry

One limitation in the present experiments is the impossibility to rule out covert language during equation verifications. Although the tasks prompted automaticity in the sense that participants had to judge the correctness of equations as fast and as accurately as possible, the extent to which participants relied on covert verbalization remains unknown. The observed response times allow the possibility that processing was not fully automatic/routinised, but that participants silently verbalized does not equal/unequal to compete the task. Future designs may be suitably extended by adding a concurrent language interference task (repeating simple digits) while English and Mandarin speakers verify equations to down-regulate the extent of language involvement, and/or conversely, by adding overt verbalization of the equations to up-regulate linguistic involvement during verifications. Another limitation concerns the absence of a significant correlation between negation-induced slowdowns in linguistic and non-linguistic contexts (Figure 2B), keeping the possibility open that other than native language-entrained factors, such as sensitivity to visual mismatches, might have played a role and could potentially predict variance in the data. In this respect, future research could benefit from combining high-level processing tasks that include different types of (non-)linguistic negation with low-level shape change detection tasks measuring modulations in visual mismatch negativity (Thierry et al., 2009).

There is a possibility that participants’ profiles, such as age, gender, literacy level, socio-economic or educational background could have influenced response times. The influence of these factors on the results cannot be ruled out, especially when considering that the Mandarin speakers were recruited from a vocational college of preschool education while the English speakers came from a university. It may be beneficial for future studies to include psychometric tests in their design to ensure that the samples are fully comparable except for the native languages. Also, the participants in the present study were predominantly females. Gender imbalance may have played some role in the variation of results, but this role is unlikely to have been a significant one as gender has not been reported to impact negation processing patterns in previous related studies. One might also wonder whether the randomly selected common geometric shapes were matched and equally understandable to participants from both language groups. Related effects are unlikely given that participants from both groups performed comparably well in the control conditions. Another step to minimize noise in the form processing cost variation due to shape-based feature differences was through the inclusion of Item as a random effect factor in all analyses. Still, future designs may find it beneficial to include a shape norming pre-test to establish equal/comparable familiarity with the shapes across groups more firmly. A further limitation is that the sample sizes in the two experiments were modest, which leaves the need for future research to recruit larger groups of participants, ideally also from different truth vs. polarity-based L1 backgrounds, to verify whether the observed patterns replicate, and whether they generalize beyond Mandarin and English speakers. On the typological level, this study may serve as a springboard to investigate the processing of negation in speakers of a variety of truth and polarity-based language systems.

Many exciting extensions and modifications can follow to advance this article’s line of inquiry. For instance, it could be turned into an experimental advantage that negative equations offer somewhat more freedom to manipulate constituent order than negative sentences do. Even though one could argue that the canonical order in equations is that the symbol appears in-between some constituent on the left side and some on the right side ▲ ≠ ■, equations with swapped orders ≠▲ ■, ▲ ■ ≠ are still comprehensible and possible to verify. Presenting various orders, for instance via a self-paced reading paradigm with one symbol shown at a time, one could track the point at which the negation symbol tends to get integrated into mental representations. If Mandarin speakers habitually attach negation globally, and integrate negation later than English speakers, one could expect faster processing with the negation symbol placed at the end of the equation. Another potentially fruitful design modifications which could help to arbitrate between the one-step vs. two-step account would be to present the negation symbol at different stimulus onset asynchronies (SOAs). If the two-step processing account holds, i.e., if individuals first simulate the affirmative and only then the negated alternative, the prediction would be that the equation symbol would interfere (delay RTs) more at later SOAs while the negation symbol would interfere more at earlier SOAs. Or, instead of RTs, a different stimulating follow-up to this study could be via a visual world eye-tracking design to monitor if participants fist fixate on ▲ = ■, or directly on ▲ ≠ ■, when they hear ‘triangle does not equal square’.

## Conclusion

Much of negation processing may be shared regardless of the languages we speak, however, this study highlights that the distinct ways in which negation is encoded in truth-based versus polarity-based systems can impact speakers’ performance even in non-linguistic tasks. This study brings new empirical evidence for linguistic relativity, showing that crosslinguistic differences in the processing of negation can extend to non-linguistic contexts. English speakers showed a reaction-time advantage over Mandarin speakers in the processing of negative equations. We attribute the reaction-time advantage of English speakers to a weaker tendency to attach negation to the statement compared with Mandarin speakers. Language-specific processing effects were observed in non-linguistic contexts for multiple areas of inquiry in previous grammatical number (Lucy, 1992; Lucy and Gaskins, 2001, 2003), motion events (Athanasopoulos and Bylund, 2013; Vanek and Selinker, 2017), time (Casasanto et al., 2004; Fuhrman et al., 2011), grammatical gender (Sera et al., 1994), color (Davidoff et al., 1999), space (Brown and Levinson, 1993; Levinson, 1996), and quantity (Gordon, 2004). However, to the best of our knowledge, this is the first study that brings empirical evidence showing that language-entrained processing routines also play an important role when processing negation beyond verbal contexts.

## Data availability statement

The datasets presented in this study can be found in online repositories. The names of the repository/repositories and accession number(s) can be found below: https://osf.io/qmgj2/.

## Ethics statement

The studies involving humans were approved by Ethics Committee of the Department of Education, University of York. The studies were conducted in accordance with the local legislation and institutional requirements. The participants provided their written informed consent to participate in this study.

## Author contributions

All authors listed have made a substantial, direct, and intellectual contribution to the work and approved it for publication.

## Funding

We gratefully acknowledge open access funding by the Faculty of Arts at the University of Auckland awarded to NV. Research reported in this article was supported by The MOE (Ministry of Education in China) Project of Humanities and Social Sciences, “A Comparative Study of Negation Processing in Chinese and English Speech Comprehension,” Grant No. 21YJC740077awarded to HZ.

## Conflict of interest

The authors declare that the research was conducted in the absence of any commercial or financial relationships that could be construed as a potential conflict of interest.

## Publisher’s note

All claims expressed in this article are solely those of the authors and do not necessarily represent those of their affiliated organizations, or those of the publisher, the editors and the reviewers. Any product that may be evaluated in this article, or claim that may be made by its manufacturer, is not guaranteed or endorsed by the publisher.
